# Incidental idiopathic intracranial hypertension

**DOI:** 10.22336/rjo.2021.37

**Published:** 2021

**Authors:** Lidia Remolí Sargues, María Isabel Soler Sanchis, Clara Monferrer Adsuara, Carolina García Villanueva, Belén López Salvador, Enrique Cervera Taulet

**Affiliations:** * 1-6 Department of Ophthalmology, Consorcio Hospital General Universitario of Valencia, Valencia, Spain

**Keywords:** idiopathic intracranial hypertension, papilledema, optical coherence tomography

## Abstract

**Objective:** Idiopathic intracranial hypertension (IIH) is a neuro-ophthalmological syndrome of unknown cause that can be vision-threatening, so an early diagnosis is crucial.

**Case report:** We reported a case of a 68-year-old asymptomatic male referred with a cataract in his right eye (OD). Best-corrected visual acuity (BCVA) was 70 letters (20/ 40) in the OD and 85 letters (20/ 20) in the left eye (OS). Ophthalmological examination revealed a significant nuclear cataract in the OD that explained the visual acuity. Fundus imaging showed a faint nasal margin elevation of the optic disc of both eyes (OU). Optical coherence tomography (OCT) revealed a sectorial retinal nerve fiber layer (RNFL) atrophy in the inferior quadrant in the OS. Nevertheless, visual field (VF) did not demonstrate defects. Neuroimaging was normal and examination of CSF revealed an opening pressure of 500 mmH2O. A diagnosis of IIH was confirmed and acetazolamide 250 mg twice daily was recommended. After 12 months of follow-up, RNFL thickness remained stable and VF did not confirm defects.

**Conclusion:** A routine eye examination was the onset of IIH in our case. Thus, the ophthalmologist played a crucial role in the early diagnosis of this syndrome. Papilledema is usually a key criterion for IIH, so after its detection, exclusion diagnosis and treatment should be initiated in order to avoid permanent visual loss.

## Introduction

Idiopathic intracranial hypertension (IIH) is a complex neuro-ophthalmological syndrome of unknown cause that can be vision-threatening and is characterized by increased intracranial pressure (ICP). It is a rare disorder with an incidence of 0.5-2 per 100.000 people per year, and most often affects women between the ages of 15 and 45 years and patients who are overweight or obese. At least 90% of patients are young females and IIH rarely affects children and men. The clinical presentation of IIH can be extraordinarily variable. Occasionally, patients are asymptomatic and are diagnosed after the detection of a bilateral optic disc swelling on a routine eye examination. Nonetheless, headaches are the most frequent onset symptom in most of the cases. Moreover, visual changes are the most common onset presentation in men. Even though headaches are truly bothersome, the most serious morbidity of idiopathic intracranial hypertension is an irreversible visual loss [**1**-**3**].

The purpose of our study was to report a male patient with an idiopathic intracranial hypertension diagnosed through a pseudopapilledema detected on a routine eye examination, in order to highlight the importance of idiopathic intracranial hypertension early diagnosis.

## Case report

**Fig. 1:**
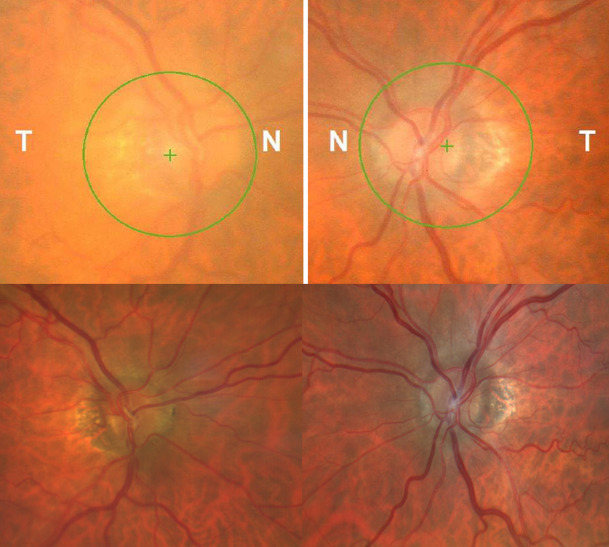
Fundus imaging at baseline (superior image) and after 12 months of follow-up (inferior image) in both eyes (OU). Fundus imaging at baseline revealed optic disc tilt, peripapillary atrophy, and a faint nasal margin elevation of the optic disc, without the presence of hemorrhages, exudates, or other signs of diabetic retinopathy or hypertensive retinopathy. After 12 months of follow-up, nasal margin elevation of the optic disc disappeared

**Fig. 2:**
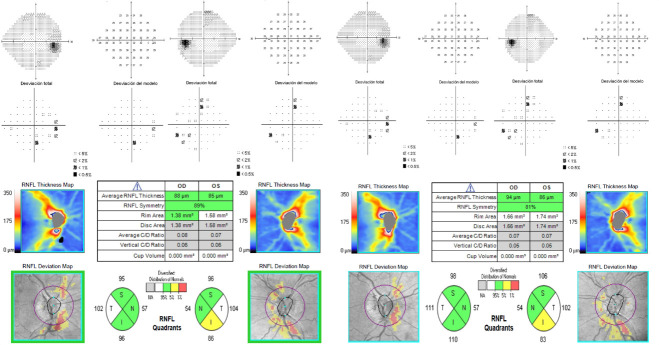
Visual field (VF) and optical coherence tomography (OCT) at baseline (left images) and after 12 months of follow-up (right images) in both eyes (OU). VF did not demonstrate defects at baseline and after 12 months of follow-up. OCT at baseline revealed a sectorial retinal nerve fiber layer (RNFL) atrophy in the inferior quadrant in the left eye (OS). After 12 months of treatment, RFNL thickness remained stable with sectorial RNFL atrophy in the inferior quadrant in the OS

A 68-year-old male was referred with a diagnosis of a cataract in his right eye (OD). He denied the presence of headache, transient visual obscurations, tinnitus, visual loss, or diplopia. He had a history of controlled arterial hypertension and type 2 diabetes treated with enalapril, nitrendipine and metformin. The patient denied taking any other medications including tetracycline, vitamins, or hormones. Body mass index was 22. Best-corrected visual acuity was 70 letters (20/ 40) in the right eye (OD) and 85 letters (20/ 20) in the left eye (OS), and Ishihara color vision test was normal. Pupillary response and extraocular motility were unremarkable. The intraocular pressures and anterior segment examination were normal, except for a significant nuclear cataract in the OD that explained the visual acuity. Posterior segment examination revealed optic disc tilt, peripapillary atrophy, and a faint nasal margin elevation of the optic disc, the rest of the fundus examination being unremarkable (**Fig. 1**). Optical coherence tomography (OCT) revealed a sectorial retinal nerve fiber layer (RNFL) atrophy in the inferior quadrant in the OS and excluded the presence of optic nerve head drusen (**Fig. 2**). Visual field (VF) did not demonstrate defects (**Fig. 2**).

**Fig. 3:**
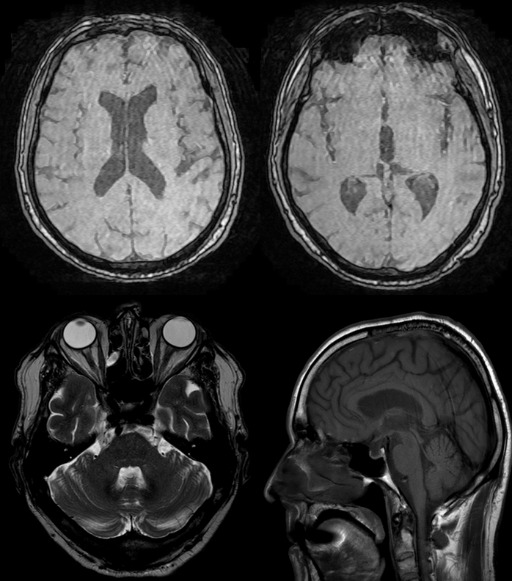
Brain magnetic resonance (MR). Brain MR showed slight degenerative changes with a subtle prominence of the cerebral sulcus. No space occupying lesions, intraparenchymal hemorrhages or ischemia areas were demonstrated

A diagnosis of low-grade bilateral optic disc swelling (Frisén grade 1) was suspected, because OCT normal RNFL thickness could not rule out the presence of a bilateral optic disc swelling due to optic disc tilt of the patient. Thus, neuroimaging, laboratory evaluation and examination of cerebrospinal fluid (CSF) were performed to exclude secondary causes of papilledema. Brain magnetic resonance (MR) and brain MR venography were normal, with no masses or structural abnormalities (**Fig. 3**). Laboratory evaluation, including complete blood count, liver function, renal function, electrolytes, and thyroid function, did not reveal alterations. Examination of CSF revealed normal cell count, glucose, and proteins, but an opening pressure of 500 mmH2O.

A diagnosis of papilledema secondary to IIH was confirmed according to diagnostic criteria of Friedman et al. [**4**] Acetazolamide 250 mg twice daily was recommended by neurologists. After 12 months of follow-up, fundus imaging returned to normal, RNFL thickness remained stable with sectorial RNFL atrophy in the inferior quadrant of the OS, and VF remained normal (**Fig. 2**). Moreover, the patient underwent cataract surgery, achieving a best-corrected visual acuity of 85 letters (20/ 20) in the OD

## Discussion

Papilledema is defined as bilateral optic disc swelling because of raised ICP, and comprises a real emergency in ophthalmology due to its possible ophthalmologic and neurologic sequelae and it can be life threatening. As soon as papilledema is confirmed, neuroimaging must be performed to rule out cerebral lesions and cerebral venous sinus thrombosis (CVST) [**1**].

Low-grade papilledema (Frisén grade 1 or 2) can be difficult to distinguish from pseudopapilledema. Pseudopapilledema is defined as an elevation of the optic disc without a real swelling of the axonal fibers, and includes several optic disc anomalies, such as hypoplastic optic disc, tilted optic disc, myelinated nerve fibers, hamartoma and drusen. The management and prognosis between both disorders are different. Therefore, OCT is necessary in order to avoid invasive procedures performed during the differential diagnosis of papilledema [**5**]. However, in our case, the diagnosis of papilledema was made due to clinical findings of fundus examination, as we considered that optic disc tilt of the patient did not allow an accurate OCT analysis. Because of that, the diagnosis of papilledema, and hence IIH, was challenging. 

Papilledema is often caused by IIH, a condition of raised ICP that predominantly affects overweight women. Clinical syndrome can include headache, transient visual obscurations, tinnitus, visual loss, diplopia, radicular pain, paresthesias, neck stiffness, arthralgias, ataxia, facial palsy, depression, and anxiety [**1**-**3**]. Asymptomatic onset is less frequent and it has been reported in children. In these cases, diagnosis is possible due to the incidental finding of a bilateral optic disc swelling on a routine eye examination [**6**]. Thus, the ophthalmologist plays an important role making an early diagnosis, as in our case. 

Secondary causes must be ruled out to confirm the diagnosis of IIH. Therefore, it is mandatory to perform laboratory evaluation and neuroimaging. Furthermore, neuroimaging is necessary to exclude space occupying lesions or ventriculomegaly, in order to perform a lumbar puncture for the confirmation of an elevated opening pressure (more than 250 mmH2O in adults) [**1**-**3**]. In atypical cases, such as children, men, non-obese patients, with progressive visual loss despite treatment, and in high-risk patients for CVST, MR venography should be performed to exclude CVST, which can have a similar presentation, and can even be life-threatening [**2**]. Because of that, MR venography was made in our patient, as we previously mentioned. 

The exact pathogenic mechanism of IIH is still unknown, although there is a clear demographic profile for this disorder. It is thought that disturbances of CSF absorption may play a crucial role. A reduction in CSF absorption at the level of the arachnoid granulations due to a stenosis in cerebral venous sinuses with secondary venous hypertension, is the most widely accepted theory. Stenosis in cerebral venous sinuses can either result from intrinsic causes or extrinsic compression by the underlying elevated ICP. Because of that, it is unclear if the elevated venous pressure is the cause or the consequence [**1**-**3**]. Since severe headache or visual loss leads to an asymptomatic onset, variable clinical presentation contributes to the knowledge about the pathogenic mechanism of IIH. Asymptomatic patients may present specific anatomic or physiologic variances (a greater number of arachnoid granulations or a better cerebrospinal fluid leak, respectively) that allow a more effective compensation of elevated ICP. Moreover, it has been reported that nutritional, electrolyte and hormonal abnormalities exacerbate the pathology, so in patients without decompensated pathologies, the symptomatic onset is less frequent [**6**].

Gondi et al. suggested that asymptomatic IIH may be a latent period of the disease in patients with better compensatory mechanisms against an increase in ICP, highlighting the importance of the early treatment in these cases [**6**]. Most patients show only mild visual field defects (such as an enlarged blind spot or arcuate defects) that regress after the reduction of the ICP, whereas some patients suffer a rapid progression of visual field defects. In addition, up to 40% of the patients with IIH can develop permanent vision loss, due to pressure induced axon loss [**1**,**7**-**8**]. Nevertheless, retinal axon loss due to increased ICP may precede clinical signs of optic disc atrophy and measurable defects in VF. Because of that, OCT becomes a powerful tool to accurately monitor structural changes in the optic nerve and retina, measuring the effectiveness of the treatment. Detection of axon loss without changes on VF may alert a coming VF loss, indicating that the treatment should be intensified [**8**-**10**]. Our patient was asymptomatic and VF was unremarkable, but detection of axon loss in OCT indicated the beginning of acetazolamide 250 mg twice daily. After that, our patient remained stable, without diminution of RNFL thickness or development of VF defects during the 12 months of follow-up.

## Conclusion

In conclusion, a routine eye examination was the onset of IIH in our case. Thus, ophthalmologists play a crucial role in the early diagnosis of this syndrome. Papilledema is usually a key criterion for idiopathic intracranial hypertension, so after its detection, exclusion diagnosis and treatment should be initiated in order to avoid permanent visual loss, the multidisciplinary approach being crucial.

**Conflicts of interest**

None.

**Informed Consent and Human and Animal Rights statement**

Informed consent has been obtained from all individuals included in this study.

**Authorization for the use of human subjects**

Ethical approval: The research related to human use complies with all the relevant national regulations, institutional policies, is in accordance with the tenets of the Helsinki Declaration, and has been approved by the institutional review board of Department of Ophthalmology, Consorcio Hospital General Universitario of Valencia, Valencia, Spain.

**Acknowledgements**

None.

**Sources of Funding**

None.

**Disclosures**

None.
